# A Slight Controversy on Antipathy of the Sexes

**Published:** 1874-04

**Authors:** 


### ANTIPATHY OF THE SEXES.

—What follows is so much better expressed than anything we can write upon the subject, that we give it the space allotted to editorial matter. The article was handed us by an intellectual and observing friend, whose statement of the truthfulness of the ideas advanced, sufficiently commends it to our notice, and that of our readers :—

A Singular but Well Expressed Opinion Relative to the Sexes—A Very Delicate Analysis of the Situation of Both Classes.—Observation goes to force upon us the unwelcome conclusion that the pretended fondness of the two sexes for one another is the great fundamental hypocrisy of the race. It would be unfair to dwell too much on the circumstance that they make one another uncomfortable in a way that men never make men nor women women, taking the fact by itself. This might be a mere result of their being different. But a review of the whole case tends to establish a general incompatibility between the two.—Things will have to alter very much if men and women are ever to get along well together. The pretence that they are dying of sheer liking for one another is not only not proved, it is disproved. Not merely is that kind of mortality wholly absent from the returns, but after all these centuries the two sexes greatly keep aloof from one another. Whenever you can get a glimpse of their true tendencies, it comes out clearly enough that men and women are domestic creatures under compulsion. Their real wish is for partial combination. All kinds of social contrivances have been tried, the real purpose of which, no matter how it may be disguised, is to separate the sexes, and so secure for each the pleasure of being only in its own society.

There is no sacrifice men will not make to get this luxury. They will support the costliest clubs, they will smoke, they will pretend any sort of recreation, from cards down to billiards, sooner than not be apart from women for a portion of their time. The like thing holds of the women in their own way. The inability of the men to stay at home allows their wives to assemble mutual clubs in their own drawing rooms, and they do so. For one club the men have, the women have hundreds —just as many as there are houses. It is all very well to decry this dominion, but of what use is that if it arises out of an incurable antipathy? The truth is, the tastes of the sexes radically differ. At home, feminine likings prevail, and there is no man that is not more or less aware that the minor arrangements and the wonderful, and to him superfluous filigree ornamentation of his house, are not for him, nor for his sex; that is the admiration of their own kind, not the opposite one, they lay themselves most out for. Men and women are in a perpetual condition of surprise, and scoff at each other's styles, both always self-complacent, and altogether omitting criticisms of their own.

The dress of the sexes utterly fails of the captivation of another. The fashionable doings of the one are mysteries to the other; for nine-tenths of the time their attire is an offence to one another. Mutual criticism on the point has not the slightest recognition, nor do the modes affect each other, save in the most rudimentary way. Each take their own course. It is not for the young ladies that the young men put on their wonderful neckties, their sleek fur collars, their astonishing jewelry, any more than it is for the male dandies the young women stay thinking and hesitating so long over the pattern of a lace or the tint of a parasol. Men never notice the pattern of the lace; they pay little heed to an umbrella, unless it is one a man is carrying. Both have in their eye those who can understand them best—their own sex. Conversation equally betrays this natural opposition. If the sexes had real respect for one another, would they indulge in those unbelievable compliments? Neither does it to those of their own kind whom they honestly like. The artificial style of talk, which is the traditionary custom of the sexes, is plainly that of creatures who do not understand each other, and have mutual suspicions. Being strange they betake themselves to compliments. A qualification in reference to the family relation has to be made.

To a man his mother is not a woman—she is a divinity; the like partly holds in a girl to her father; and brothers and sisters are not of any sex. But get outside of this non-sexual circle, and the antipathy quickly comes into play. Boys nearly hate girls, and the feeling is returned; old men care nothing for women of any age, except as nurses; old women creep together. It is only during the central portion of life that the sexes can be said to be civil to one another. In fact, if nature had not forced men and women to love each other during that portion willynilly, and give them that incredible and perplexing bribe of children, it is doubtful whether they would have any mutual liking. Love is all that exists between them. The score of other feelings of sympathy, of appreciative respect, of rational emulation which men have for men, and women for women, neither sex has for the other.—*Pall Mall Gazette.*

—There's not a shadow of truth in this article, from beginning to end. It is a *lie-bel* on both men and women, and I'm ashamed of you, sir, for endorsing it.

YOUR WIFE.

Our wife, who occasionally acts as our proof reader, returned the proof sheet containing the above article, with the endorsement as given. As she has never had an opportunity for appearing editorially in the BISTOURY, we cheerfully accord her the privilege, notwithstanding she is likely to be astonished at her sudden debut. As she is a very sensible and observing woman, we are almost persuaded to qualify our quasi endorsement of the article in controversy, by simply asserting that there are many grains of truth in it, but out of respect to wife's opinion, we think, or probably had *better* think, (as we abhor curtain lectures) that the article in all its bearings is not in exact accordance with our experience. Upon reflection, we are sure it is not; and, when a marked copy reaches the wife of our friend who had the audacity to get us into this difficulty, we are inclined to the opinion that his ears will ring with conjugal upbraidings. Having a "doctor" in his family, he must needs take his own physic.—There, we wonder whether we have fairly gotton out of that? If not, friend Charles, come to our relief and defend your article!

